# Efficacy of Left Prefrontal Transcranial Direct Current Stimulation in Improving Negative Symptoms and Acutely Enhancing Attention Functions

**DOI:** 10.1192/j.eurpsy.2025.1693

**Published:** 2025-08-26

**Authors:** H. Noyan, G. Eskikurt, A. Verkhovets, N. Zeren, H. Yeşilkaya, A. Üçok

**Affiliations:** 1 Department of Psychology, Ankara University, Ankara; 2 Department of Psychology, Istinye University Faculty of Humanities and Social Sciences; 3 Master Degree Programs of Clinical Psychology , Beykoz University Institute of Graduate Programs; 4 Department of Psychiatry, Istanbul University Istanbul Medicine Faculty; 5 Bakirkoy Training and Research Hospital for Psychiatry, Istanbul, Türkiye

## Abstract

**Introduction:**

Negative symptoms and cognitive deficits in schizophrenia (SZ) significantly affect patients’ quality of life and functionality, but show limited response to antipsychotic treatment.

**Objectives:**

The present study aims to investigate the acute (n=32) and one-month (n=25; active-tDCS:13 vs. sham-tDCS:12) effects of repeated transcranial Direct Current Stimulation (tDCS) on negative symptoms and executive attention in recent-onset SZ.

**Methods:**

In this study, 32 clinically stable SZ patients (age:24.7±4.5, 65% male) with a disease duration under 5 years were included in a single-blind, randomized sham-controlled trial. Patients received 10 sessions of either active-tDCS (n=17) or sham-tDCS (n=15) (anode: left dorsolateral prefrontal cortex, DLPFC; cathode: right orbitofrontal region) at 2 mA for 20 minutes, twice daily, across 5 consecutive days and were followed up after the 10th tDCS on day 5 (acute effect), and at weeks 2 and 4. Pre- and post-tDCS assessments included the Brief Negative Symptom Scale (BNSS), Brief Psychiatric Rating Scale (BPRS), Calgary Depression Scale for SZ (CDSS), Global Assessment of Functioning (GAF), Clinical Global Impression Scale, Verbal Fluency Test (VFT), and the Penn Computerized Neurocognitive Battery’s Letter N-Back and Continuous Performance Test (CPT). One-way ANCOVA was used to assess between-group changes over time, controlling for pre-tDCS measurements, while mixed-design ANOVA explored time × tDCS-group interactions, followed by repeated measures ANOVA to assess within-group effects. Pairwise comparisons over time within each group were examined using a post-hoc Bonferroni test.

**Results:**

The active-tDCS group showed significant acute improvements in Avolition-Apathy (AA) (F_(1,29)_=13.55, *p*<0.001, p*η²*=0.319) and Expressive Deficit (EXP) (F_(1,29)_=4.66, *p*=0.039, p*η²*=0.138) domains, BNSS-total (F_(1,29)_=25.12, *p*<0.001, p*η²*=0.464), BPRS-General Psychopathology (F_(1,29)_=19.68, *p*<0.001, p*η²*=0.404), CDSS (F_(1,29)=_8.16, *p*=0.008, p*η²*=0.22) scores, VFT-phonemic fluency (F_(1,29)_=11.98, *p*=0.002, p*η²*=0.292) and CPT (F_(1,29)_=5.29, *p=*0.029, p*η²*=0.154) performances compared to sham-tDCS. Time-group interactions were significant in BNSS-AA domain (F_(1,21)_=16.44, *p*<0.001, p*η²*=0.701), BNSS-total (F_(1,21)_=15.77, *p*<0.001, p*η²*=0.693), and BPRS-General Psychopathology (F_(1,21)_=6.42, *p*=0.003, p*η²*=0.479). Following the mixed-design ANOVA, repeated measures analyses showed significant score decreases over time in the active-tDCS, while the sham group showed no significant changes or a slight increase (only for BNSS-total scores) (Fig. 1).

**Image 1:**

**Figure:**
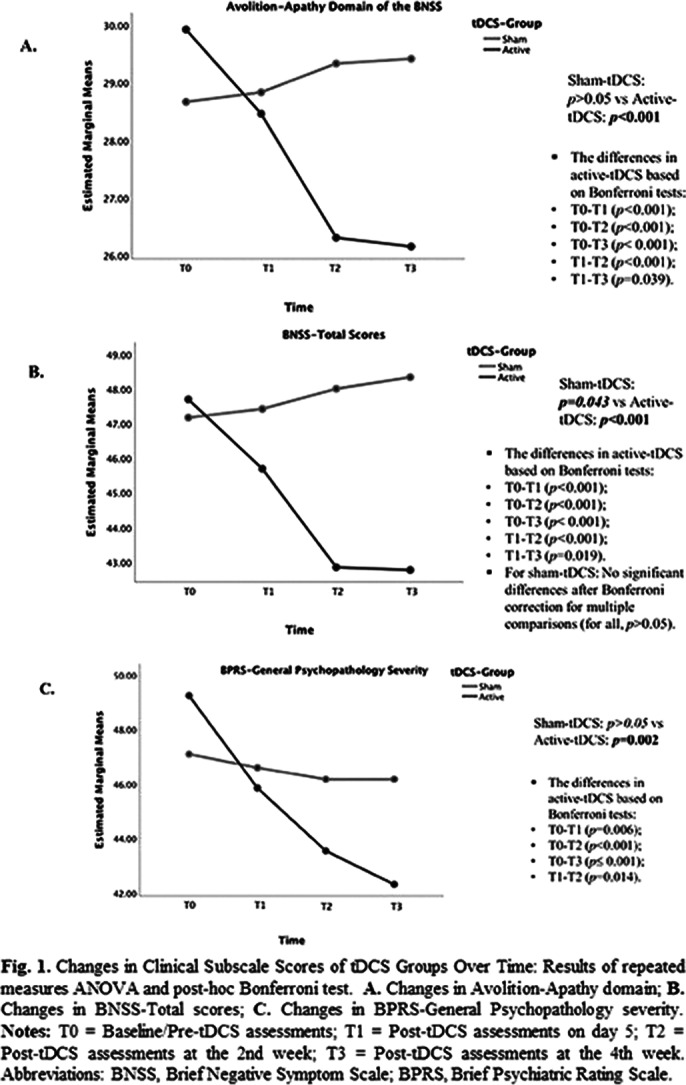

**Conclusions:**

The present results suggest that anodal tDCS over the left DLPFC may be effective in alleviating negative symptoms, reducing general psychopathology severity, and acutely enhancing complex attention functions and working memory in recent-onset SZ.

**Disclosure of Interest:**

None Declared

